# Superactivation of p53 Using Drug Combinations and New Insights into p53 Functions and Anticancer Therapy

**DOI:** 10.3390/ijms27208964

**Published:** 2026-10-09

**Authors:** Marek Rusin, Małgorzata Krześniak, Barbara Łasut-Szyszka

**Affiliations:** Laboratory of Preclinical Models, Maria Skłodowska-Curie National Research Institute of Oncology, Gliwice Branch, 44-101 Gliwice, Poland; malgorzata.krzesniak@gliwice.nio.gov.pl (M.K.); barbara.lasut-szyszka@gliwice.nio.gov.pl (B.Ł.-S.)

**Keywords:** p53, death receptors, innate immunity, drug synergy, *DRAXIN*, *SLAMF7*, *SOCS1*

## Abstract

The p53 tumour suppressor protein exerts its influence on cells by regulating the expression of more than one thousand genes. To study p53 functions, cells are treated with various p53 activators, mostly established or experimental anticancer drugs. Some of these substances strongly collaborate in the activation of p53, which is manifested by a pronounced increase in the number of p53 molecules phosphorylated at both the amino and carboxyl terminus, a sign of p53 activation. Another manifestation of this collaboration is an increase in the number of upregulated genes. A good example of apparent synergy is the treatment of cells with actinomycin D and nutlin-3a, although other drug combinations show similar effects. The molecular mechanism underlying this collaboration is not precisely known. This superactivation of p53 has potential therapeutic applications. For example, it strongly sensitises cancer cells to apoptosis induced by FASLG, a ligand of the FAS death receptor. Moreover, superactivated p53 enables the identification of new p53 target genes, which helps us to better understand poorly studied p53 functions, such as the regulation of innate immunity, modification of cell microenvironments, removal of apoptotic bodies, tissue repair, and hormone production. However, innovative drug combinations targeting the p53 pathway have yet to be successfully translated into clinical practice.

## 1. Introduction

We start this review with a short description of p53 activity as a transcription factor. Then we focus on the molecular and biological consequences of p53 superactivation by collaborative action of actinomycin D and nutlin-3a (A+N). In the next sections we present plausible functions of p53, which were deduced based on the analysis of the genes activated by A+N. Therefore, the reference to A+N combination will interweave through these sections. Next, we discuss whether the activation of p53 by A+N reflects a natural stress factor. We end with the description of other drugs, which collaborate in the activation of p53.

## 2. p53 as an Exceptional Activator of Gene Expression

The p53 tumour suppressor protein is a transcription regulator, which binds to its 20 bp cognate DNA sequence within gene promoters or enhancers and induces the expression of more than one thousand genes [1,2,3]. This conservative census of direct p53 targets is based on high-throughput studies measuring both gene activation in response to stress and p53 binding in the vicinity of relevant genes [2]. As a gene regulatory protein, p53 is exceptional because its DNA binding site is two times longer than the binding sites of the overwhelming majority of other transcription factors [3]. This has consequences, which are discussed later. In addition to activating gene expression, p53 can also repress transcription. However, this function is mediated predominantly through indirect mechanisms. For instance, p53 directly activates the gene *CDKN1A* for the p21 protein, which, in turn, through a cascade of events, shuts down the genes involved in DNA replication and other functions essential for cell division [4]. Among the genes activated by p53 are two major groups—genes activated in most cell types by most stress factors (approximately 100 genes) and genes stimulated in a cell- or stress-specific manner [5]. Considering the estimated number of genes directly activated by p53 [2] it can be inferred that the bulk of p53 targets is regulated in a stress- or cell-specific manner. Cell specificity may be related to chromatin modifications [5]. In some cell types, the chromatin containing p53-activated genes is so tightly condensed that p53 cannot initiate gene transcription. In others, some genes are constitutively highly expressed and p53 cannot further stimulate their transcription [6]. It is also plausible that in certain cells, p53 does not stimulate some genes due to a lack of necessary cofactors or enzymes, which post-translationally modify p53 [7]. On the other hand, p53 can not only actively initiate transcription but also promote histone modification and nucleosome eviction. Thus, the regulation of transcription by p53 is multilayered [8].

The target genes differ in the degree of their activation following p53 stimulation. Their expression may increase from several-fold to several hundred-fold or even more [9,10]. The magnitude of gene induction is likely influenced by the basal level of gene expression. It can be speculated that genes with high basal expression will generally show smaller relative increases, whereas genes expressed at very low or undetectable levels can display very large induction following p53 activation. Presumably, the impact of p53 on cell physiology will be stronger through genes with a high fold change in expression. The fold change may also be governed by factors such as the strength of p53 binding to its cognate DNA sequence or to other proteins that collaborate with p53 in the stimulation of transcription.

The best known mode of p53 regulation is linked to its abundance within a cell. Under normal growth conditions, the level of p53 is low due to its fast degradation in proteasomes, induced by ubiquitination catalysed by MDM2 ubiquitin ligase, which is the major negative regulator of p53 (reviewed by Brown et al. [11]). Moreover, MDM2 conceals and inhibits the transactivating domain (TAD) of p53, located at its amino terminus [12]. MDM2 performs this concealment together with its partner—the MDMX protein. The MDM2-p53 interaction is prevented when various kinases activated by stress factors phosphorylate the amino-terminus of p53. This prevents its degradation and leads to a strong accumulation of p53 as well as to a better interaction between this protein and other transcription activating factors. For instance, the phosphorylation of several residues on the N-terminus of p53 increases its binding to the p300 transcriptional co-activator by almost 100-fold and significantly reduces its binding to MDM2. The relative affinities of MDM2 and p300 for the p53 transcriptional activation domain can be changed by up to three orders of magnitude by phosphorylation [13]. The measurements of affinities between the p53 TAD and the relevant domains of MDM2 and p300 were performed in vitro using a method called fluorescence anisotropy titration. The effects of p53 phosphorylations at Ser15, Thr18, Ser20, Ser33, Ser37, Ser46 and Thr55 on the binding to MDM2 or p300 were assessed. Phosphorylations of individual amino acids and all seven residues were assessed. The interaction between TAD and p300 increased strongly (7-fold) upon phosphorylation of Thr18; however, the interaction was much stronger (80-fold) when all seven amino acids were phosphorylated. Binding of MDM2 to TAD was significantly reduced upon phosphorylation of Thr18 (7-fold), and was reduced even more strongly (24-fold) when all tested amino acids were phosphorylated [13]. Thus, it can be inferred that the greater the number of phosphorylated amino acids in TAD, the higher the affinity to transcriptional co-activators and the lower the affinity for MDM2. Collectively, these changes lead to an increased expression of p53-regulated genes (reviewed by Brown et al. [11]). The interaction between p53 and MDM2 can also be prevented by other mechanisms. For instance, the overstimulation of cell growth and division by oncogene activation results in MDM2 sequestration by the p14ARF protein [14]. A similar mechanism is activated when actinomycin D inhibits the transcription of ribosomal RNA—some ribosomal proteins bind to MDM2 and prevent it from inactivating p53 [15]. However, this is not the only mechanism by which actinomycin D activates p53. This drug also promotes p53 phosphorylation at the amino-terminus. Actinomycin D induces the accumulation of p53 with phosphorylated serines 9, 15, 20, 37 and 46 [16]. The question is whether the stress induced by actinomycin D activates kinases, which phosphorylate p53, or whether MDM2 sequestration by ribosomal proteins enables easy access of constitutively active kinases to the amino terminus of p53. With these questions in mind, we decided to expose cancer cells to both actinomycin D and nutlin-3a, which is the first of many experimental drugs designed to block the p53-binding pocket within the MDM2 protein preventing the interaction between these two molecules [3] (Figure 1). We hypothesised that actinomycin D would activate some kinases that phosphorylate p53 (Figure 1), while nutlin-3a, by preventing the interaction between MDM2 and p53, would give the activated kinases unhindered access to p53, allowing for the extensive phosphorylation of its TAD (Figure 1). This hypothesis was based on observations showing that actinomycin D can promote the phosphorylation of p53 [16], although the exact kinase responsible was not identified. The plausible role of nutlin-3a in facilitating p53 phosphorylation was based on observations that this molecule blocks the interaction between MDM2 and p53 [3] and that MDM2 conceals that transcriptional activation domain of p53, which is the major target of activating phosphorylation [12]. Blocking the blocker should, in principle, facilitate the actinomycin D-activated kinases in extensively phosphorylating p53 (as explained in the legend of Figure 1). In this way, we hoped to generate superactivated p53. Thus, the plausibility of synergy in the activation of p53 between actinomycin D and nutlin-3a was deduced based on the known mechanisms of action of these two molecules.

## 3. Actinomycin D and Nutlin-3a Induce Superactivation of p53

The co-treatment of various cancer cell lines and normal human fibroblasts with actinomycin D and nutlin-3a revealed a very strong apparent synergy between the two compounds in the induction of the phosphorylation of various amino acids on both the amino (Ser15, Ser33, Ser37, Ser46) and carboxyl (Ser392) terminus of p53 (Figure 2A). Other residues (Thr18, Ser20, Thr55) modulating the interaction between TAD of p53 and MDM2 or p300 [13] were not examined in cells exposed to A+N [10,18,19,20]. This result was consistent with our hypothesis, although we did not identify the enzymes responsible for these phosphorylations. Two kinase inhibitors, CHIR-98014 and C16, attenuated the phosphorylation of p53 induced by A+N. However, when we downregulated the expression of the known targets of these inhibitors—GSK3A, GSK3B and PKR—the phosphorylation status of p53 did not change [19,20]. Thus, the kinases responsible for p53 phosphorylation following exposure to A+N have not been identified. The results of experiments with CHIR-98014 were particularly interesting [20] because this inhibitor almost completely blocked the expression of a subset of genes activated by A+N, like *CASP1*, *H19*, *CRYAB*, and *STING1,* but had no influence on the activation of well-known p53 targets such as *FAS*, *PMAIP1*, *BBC3*, and *CDKN1A* (p21). Thus, the kinase inhibited by CHIR-98014 enables the activation of only a subgroup of p53-regulated genes. This suggests that the activity of particular kinases determines the set of genes stimulated by p53.

Co-treatment with actinomycin D and nutlin-3a induced a substantial accumulation of p53 molecules with phosphorylated serine 46, and these compounds considerably collaborated in phosphorylation on this residue (Figure 2A), which is considered a marker of strong p53 activation associated with the induction of apoptosis [21]. In spite of this, the frequency of apoptotic cells in the examined cell line was low [20]. Some cells died by apoptosis, which was visible by the activation of caspase-3. Some cells appeared to have died by necrosis, which was visible in their morphology, though the overall death frequency was low [18,19,20]. The other surprise was that not all tested p53-regulated genes were upregulated by actinomycin D and nutlin-3a in a collaborative fashion. This suggests that p53-regulated genes vary in their response to the activation mode of p53 (see below). Yet, the apparent synergy between actinomycin D and nutlin-3a in the activation of some genes was so remarkable that this method was employed to search for novel, p53-regulated genes [22,23]. This drug combination had one big advantage, namely, it caused a strong activation of p53 by engaging two signalling pathways converging on this protein. This contrasts with strong activation induced by extensive damage to the cells by a high dose of ionising radiation (IR) or by a high concentration of DNA-damaging compounds, which, in addition to the activation of p53, induce much collateral damage, triggering other stress-response systems.

A systematic search for genes upregulated by actinomycin D and nutlin-3a, administered alone and in combination, was performed by RNA sequencing using the A549 lung cancer cell line as a model. Additionally, for comparison, the cells were treated with camptothecin, which also strongly activates p53, although to a lesser degree than A+N [19]. Moreover, three other cell lines were exposed to A+N: NCI-H460 (lung cancer), U-2 OS (osteosarcoma), and A375 (melanoma). This enabled a comparison of gene expression in cells originating from different tissue backgrounds. The transcriptomic data were combined with proteomic data obtained using mass spectrometry performed on cell lysates and a conditioned culture medium from A549 cells exposed to A+N. This allowed the authors to determine if the newly discovered p53-regulated genes actually translate into proteins, and which of the corresponding proteins can be readily detected in cell lysates and the culture medium [10]. The main finding was that the possible synergy between actinomycin D and nutlin-3a was most evident in the number of strongly upregulated genes (at least four-fold) (Figure 2A). Exposure to nutlin-3a activated 94 genes, actinomycin D induced 133 genes, while treatment with the two substances strongly upregulated 587 genes. The fact that A+N upregulates over six times more genes than a typical p53 activator, like nutlin-3a, enabled the identification of new p53 targets. Interestingly, the majority of these targets are also upregulated by camptothecin, which strongly (at least four-fold) activates 388 genes. Thus, many genes (314) upregulated by the A+N combination are also activated by the potent anticancer alkaloid, which gave rise to two anticancer drugs, topotecan and irinotecan. A closer inspection of the upregulated genes confirmed the initial observation that actinomycin D and nutlin-3a do not cooperate in the upregulation of some of the well-known p53 target genes like *MDM2*, *ZMAT3* and *FAS* (Figure 2B). However, in the majority of upregulated genes, actinomycin D and nutlin-3a showed either an additive (*FBXO2*, *BBC3*) or an apparently synergistic effect (*INKA1*, *STING1*, *ISG15*, and *MAFB*). In some genes, e.g., *MAFB*, the synergy is striking. Thus, p53 target genes show heterogeneity in response to p53 activation. Some genes, e.g., *MDM2* and *FAS*, respond in a similar fashion, regardless of the amount of p53 or its post-translational modifications; however, the response of other genes, e.g., *STING1* and *ISG15*, closely reflects the amount of total p53 and the amount of its post-translationally modified forms. Thus, it appears that every p53 target gene responds in its own way to the activation of p53 and its post-translational modifications. In other words, the nature of p53 activation may influence which subsets of p53-dependent biological programmes become engaged. While the canonical p53 responses, such as MDM2 or FAS induction, appear to be relatively robust and less dependent on the extent of p53 activation, other programmes may require stronger p53 activation or specific post-translational modifications. This may be particularly relevant for p53-regulated genes involved in immune-related processes, such as *STING1* and *ISG15*, suggesting that high-level or qualitatively distinct p53 activation may preferentially reveal specific functional outputs of the p53 response.

The question is what mechanism is responsible for the activation of so many genes by A+N. Part of the answer may be related to post-translational modifications of p53 and the total amount of p53. The number of p53 molecules with phosphorylated serines 46 and 392, and the total number of p53 molecules, is significantly higher in cells exposed to A+N compared to cells exposed to actinomycin D or nutlin-3a alone (Figure 2A). How do the post-translational modifications increase the number of genes activated by p53? There is an intriguing hypothesis: p53 acts as a tetramer, and each monomer binds to the pentanucleotide fragment of the 20 bp consensus sequence. When interactions between p53 monomers become stronger due to post-translational modifications, the p53 tetramer is able to bind to additional sites that show a weaker match to the consensus sequence. This hypothesis is supported by observations that artificially engineered amino acid substitutions within p53, which promote the cooperative binding between monomers, enable the p53 tetramer to bind and strongly activate additional genes, e.g., those involved in the induction of apoptosis [24]. Some post-translational modifications of p53 promote tetramerisation, e.g., phosphorylation of Ser392 [25]. Interestingly, the number of p53 molecules with this modification significantly increases following treatment with A+N, and actinomycin D strongly collaborates with nutlin-3a in this process [19].

## 4. DRAXIN as an Intriguing p53 Target Gene

As mentioned earlier, we exploited treatment with actinomycin D and nutlin-3a to search for novel p53-regulated genes. Initially, we employed the candidate gene approach [19,22,23], followed by a more systematic search using RNA sequencing [10,20]. We indeed identified many novel p53-regulated genes that have not been identified as such in the context of the existing literature. Many genes activated by A+N encode innate immunity proteins [10,19,20] and the regulators of the Wnt/β-catenin pathway [10]. There is also a large group of genes that drive the production of extracellular proteins [10]. Thus, p53 not only induces the expression of proteins promoting cell cycle inhibition, apoptosis, autophagy, changes in metabolism etc., but also modifies the cell microenvironment. An intriguing gene that impacts both the Wnt/β-catenin pathway and extracellular space is *DRAXIN* [10,23]. Actinomycin D and nutlin-3a strongly collaborate in the activation of this gene, though it is also activated by these compounds acting independently. The activation of DRAXIN by p53 is intriguing because, under normal conditions, it is expressed primarily in the developing central nervous system, acting as a chemorepulsive guidance cue for growing axons [26]. Using A+N, we demonstrated that it can be activated by p53 in cell lines derived from lung cancer and osteosarcoma. Mass spectrometry and Western blotting demonstrated that this protein is actually present in the culture medium of cells exposed to A+N. The role played by DRAXIN outside the developing central nervous system is a complete mystery. It was shown that it can inhibit the Wnt/β-catenin pathway [27], which is oncogenic when hyperactive [28]. Thus, DRAXIN may be part of the tumour-suppressive mechanism of p53. However, it is also possible that DRAXIN plays a more subtle role in cancer biology. There is an intriguing possibility that DRAXIN inhibits tumour infiltration by neurons. Nerve cells were shown to fuel tumour growth through chemical and electrical signals [29]. Thus, by activating DRAXIN, p53 may prevent tumour infiltration by neurons. However, this hypothesis can only be tested by using advanced models involving the growth of complex organoids. There is preliminary evidence that DRAXIN overexpression may contribute to the pathology of Alzheimer’s disease [30]. The role of DRAXIN as a mediator of p53-dependent functions warrants further investigation.

## 5. Actinomycin D and Nutlin-3a Collaborate to Sensitise Cells to Death Receptor-Induced Apoptosis

While the role played by DRAXIN induced by p53 is speculative, other biological effects of p53 activation by A+N are better defined. As mentioned earlier, despite the strong upregulation of many proapoptotic genes, the frequency of apoptotic cells in the population exposed to A+N is low (at least in some cell types). One of the genes activated by actinomycin D and nutlin-3a encodes the death receptor called FAS (Figure 2B). Stimulation of FAS by its ligand (FASLG) initiates a signalling cascade involving caspase-8, caspase-10, and the executioner caspase-3, ultimately culminating in apoptosis. We hypothesised that A+N would sensitise cells to FASLG-induced apoptosis. Indeed, we observed that more than 99% of cells pretreated with A+N and subsequently exposed to FASLG for 5 h died [31]. This effect was critically dependent on the presence of p53 (Figure 3). In p53-deficient cells, FASLG-induced apoptosis did not differ from background visible in untreated cells. Although FAS receptor expression was very similar in cells exposed to actinomycin D, nutlin-3a, or to both compounds (Figure 2), these compounds strongly collaborated in the induction of cell death (Figure 3A). Thus, the molecular mechanism of apparent synergy in this model remains to be determined [31].

Actinomycin D and nutlin-3a collaborated in the upregulation of many relevant proapoptotic genes (*CASP6*, *CASP10*, *APAF1*, *PMAIP1*, and *BIK*), but no clear-cut synergy was observed. Thus, the molecular mechanism underlying the sensitisation may be less obvious than the upregulation of well-known proapoptotic genes. One hypothetical mechanism involves the change in the lipid composition of the plasma membrane, which promotes the formation of membrane rafts associated with FAS receptor aggregation and the amplification of the proapoptotic signal [34]. Treatment with A+N activates some genes, e.g., *SMPDL3B* [10], whose protein products are able to change the composition of the plasma membrane [35]. Another intriguing possibility is suggested by the analysis of transcriptomic data. Actinomycin D and nutlin-3a collaborate in the upregulation of the *INKA1* gene, which encodes a potent endogenous kinase inhibitor (Figure 2 and Figure 3B). One of its targets is PAK4 kinase [32]. PAK4, in turn, can inhibit apoptosis early in the caspase cascade by antagonising the activation of the initiator caspase 8 [33]. Thus, in principle, INKA1, by inhibiting the inhibitor of caspase 8, may promote FASLG-induced apoptosis. This hypothesis requires experimental testing, but we mention it to demonstrate that the mechanism of sensitisation to FASLG may be more complicated than the simple upregulation of the best-studied elements of the signalling pathway leading from the death receptor to executioner caspases. Regardless of the mechanism, this effect has potential clinical applications because the induction of cancer cell death is very strong and normal human fibroblasts are less sensitive to this treatment. Interestingly, in this study, we also found that a similar sensitising effect can be achieved by different drug combinations, e.g., actinomycin D can be substituted with etoposide, and nutlin-3a can be substituted with newer inhibitors of the p53-MDM2 interaction, like idasanutlin or RG7112. The fact that the sensitising effect can be reproduced using different combinations of DNA-damaging agents and MDM2 inhibitors suggests that the observed response is not a drug-specific phenomenon but rather reflects the activation of a specific p53-dependent state. The strong dependence on the presence of p53, together with the apparent all-or-none nature of the response, indicates that p53 may function as a molecular switch controlling this apoptotic programme rather than merely modulating it quantitatively. This suggests the existence of a specific p53-dependent mechanism that selectively enables tumour cells to become highly sensitive to FASLG-induced apoptosis. In conclusion, this method of inducing apoptosis has three remarkable features—strong collaboration between actinomycin D and nutlin-3a in the sensitisation to FASLG, critical dependence on the presence of p53 with an all-or-none effect, and the almost-synchronous appearance of apoptotic cells following the addition of FASLG. At the level of individual cells, cell death, e.g., apoptosis, follows an all-or-none principle (cells either remain alive or die). However, in the case of p53 superactivation, virtually all p53-proficient cells in the population pre-exposed to A+N quickly die after the addition of FASLG. It appears that the induction of apoptosis may be a complex, multistep process involving the priming of cells through the activation of a set of proapoptotic genes. The final commitment to apoptotic cell death may occur upon exposure to an apoptotic trigger, such as FASL in our case, which alone is insufficient to induce widespread apoptosis but can effectively trigger cell death in cells that have been appropriately primed. In other words, the activation of p53, even when very strong, *per se* may not be enough to induce widespread apoptosis unless a specific trigger is present. Currently, we are testing the efficiency of this drug combination in inducing apoptosis and the inhibition of tumour growth in mouse models.

## 6. Actinomycin D and Nutlin-3a Cooperate in the Stimulation of Innate Immunity

Analysis of transcriptomic data revealed that the A+N combination activates genes encoding proteins that make cancer cells better targets for natural killer (NK) cells. These innate immune cells recognise stress factors on the surface of their targets and kill them using FAS ligands or a caspase-activating protein called granzyme, injected into the cells destined for destruction. In addition to genes identified by others, e.g., ULBP2 [36,37], A+N induced the expression of *SLAMF7* and *NCR3LG1* in a p53-dependent manner [38]. SLAMF7 is particularly intriguing because, so far, its expression has been only found on the surface of immune cells. SLAMF7 is also upregulated in a p53-dependent manner by camptothecin [38]. Moreover, this protein is a target for a therapeutic antibody called elotuzumab, used to treat multiple myeloma, which expresses high levels of SLAMF7 [39]. Pre-treatment of cancer cells with drugs that induce SLAMF7 expression creates the possibility of extending the use of elotuzumab to neoplasms other than multiple myeloma.

While A+N induces the expression of proteins that activate NK cells, the killing effect is not as spectacular as in the case of the FAS ligand, perhaps because the interaction between cancer cells and NK cells in vitro lacks some important microenvironmental cues. There is clear cooperation between actinomycin D and nutlin-3a in the sensitisation of cancer cells to elimination by NK cells, but the effect of p53 is not as clear-cut as in the case of the FAS ligand. Apparently, some p53-independent mechanisms also play a role in this sensitisation. However, it is indisputable that p53 promotes the expression of proteins that serve as activators of NK cells [36,37,38].

Another biological outcome of treatment with A+N is the modulation of interferon signalling. Treatment with A+N induces the expression of some interferon-stimulated genes despite the absence of evidence for autocrine interferon stimulation, e.g., by the lack of STAT1 phosphorylation [19]. These initial observations were confirmed by more systematic pathway enrichment analysis performed on the group of upregulated genes detected using RNA sequencing [10]. There was also cooperation between actinomycin D and nutlin-3a in the upregulation of the *SOCS1* gene, which encodes a negative regulator of STAT1, a transcription factor that is a major mediator of gene activation induced by interferons, both type I (e.g., interferons α and β) and type II (interferon γ) [19]. Indeed, the activation of p53 by A+N attenuates the phosphorylation of STAT1, induced by interferon-α1, but it did not uniformly inhibit the expression of genes stimulated by this cytokine. Likely, the attenuation of STAT1 phosphorylation by p53 was small when compared with the large increase in the phosphorylation induced by interferon-α1. In contrast to this, the outcome of p53 activation by A+N and the exposure of cells to interferon-γ was more clear-cut. p53 and this cytokine apparently synergised in the upregulation of some genes, such as *CASP1*, *IFIT1* and *IFIT3*, suggesting that these two signalling pathways cooperate in cell physiology modulation [40].

The regulation of *SOCS1* expression by p53 is unusual. In high-throughput experiments, this gene was found to be either upregulated (12 studies) or downregulated (9 studies) in response to p53 [9]. In A549 lung cancer cells, the *SOCS1* gene is strongly upregulated by A+N in a p53-dependent manner [19]. Interestingly, these cells exhibit low constitutive expression of *SOCS1*. In contrast, U-2 OS osteosarcoma cells show high constitutive expression of this gene, which is downregulated in a p53-dependent manner, but only in cells exposed to nutlin-3a. Considering that p53 binding near the *SOCS1* gene in A549 cells was found in some ChIP-Seq studies, and p53 binding near SOCS1 was not detected in U-2 OS cells exposed to nutlin-3a ([40] and refs therein), it can be concluded that p53 directly stimulates the expression of *SOCS1* in A549 cells, and it indirectly represses this gene in U-2 OS cells. Thus, *SOCS1* may be a good example of a gene whose regulation by p53 is context-dependent and varies according to both the cellular context and the activating factor.

## 7. Can p53 Facilitate the Clearance of Apoptotic Bodies?

Detailed analysis of the functions of genes activated by A+N points to an interesting mechanism by which p53 may deal with the remnants of apoptotic cells. p53 not only promotes the expression of proteins, which directly induce apoptosis, but likely also prepares cells to be efficiently engulfed by scavengers like macrophages. One of the genes upregulated by A+N is *MFGE8* [10]. Although the increase is only approximately three-fold, the gene encodes the protein that binds to phosphatidylserine exposed on the surface of apoptotic bodies, and facilitates their recognition and engulfment by macrophages [41]. A complementary function is played by another gene, *XKR8*, whose expression was increased approximately four-fold by A+N [10]. The protein encoded by this gene is called scramblase, and it mediates the transition of phosphatidylserine from the inner to the outer leaflet of the plasma membrane. During apoptosis, the XKR8 protein is activated by caspase-3 [42]. Apparently, A+N, in preparation for apoptosis, promote the expression of this protein, which is responsible for phosphatidylserine exposure on the cell surface, which, in turn, is bound by MFGE8 and likely other proteins that enable macrophage engulfment. Apparently, p53, by promoting the expression of XKR8 and MFGE8, enables the quick removal of apoptotic bodies from tissues. This illustrates that p53 activates the apoptotic programme in a comprehensive manner, affecting both the induction of apoptosis and the removal of apoptotic bodies from tissues.

## 8. p53 and Tissue Repair

Widespread apoptosis of cells induced by severe stress factors may significantly alter local tissue architecture. Thus, when stress has subsided, the correct cell arrangement must be restored. Hence, it is plausible that p53 not only induces the expression of direct activators of apoptosis and promotes the expression of proteins that facilitate the removal of apoptotic bodies, but may also help in the restoration of normal tissue architecture after cells cease to die. This proposed function of p53 is speculative and more difficult to study, because it requires more advanced animal models or multicellular organoid cultures. Many of the 587 genes upregulated at least four-fold by A+N treatment encode extracellular proteins. We have already mentioned DRAXIN, but there are other upregulated genes that encode molecules involved in angiogenesis and remodelling of the extracellular environment. For example, *ECM1* and *ANGPT4* promote angiogenesis [43,44]. Increased expression of proangiogenic factors may appear to be counterintuitive for a tumour suppressor such as p53; however, it is possible that their transient increased expression could be associated with post-apoptotic tissue repair. Equally counterintuitive is the upregulation of KIT ligand (KITLG) expression by A+N. This protein stimulates the oncogenic KIT receptor. KITLG is also known as a stem cell factor. Its upregulation by p53 has previously been reported [1]. We hypothesise that KITLG activated by p53 may promote the local proliferation of stem cells to rebuild tissue following cell elimination by apoptosis. ADAMTS7 is also a gene strongly upregulated by A+N. It encodes metalloprotease, which degrades the components of the extracellular matrix. Unfortunately, the biological functions and substrate targets of ADAMTS7 remain poorly studied, limiting our comprehension of its roles in both health and disease [45]. Its activation by A+N suggests that it may be a mediator of a p53-dependent reconstruction of the extracellular matrix. Remodelling of the extracellular matrix can also be mediated by the FBLN5 protein, which is essential for elastic fibre formation [46]. LAMC3 is a component of basement membranes [47]. In conclusion, our published [10] and unpublished transcriptomic and proteomic data indicate that A549 cells exposed to A+N secrete a plethora of proteins involved in extracellular matrix remodelling. Further details on this poorly publicised function of p53 can be found in a recent review [48]. However, it is very difficult to predict the net biological effect of the secretion of p53-regulated proteins based on the functions of individual molecules, as some of these factors may exert opposing or counteracting effects. The overall biological outcome can therefore only be determined in more physiologically relevant models, such as organoid or in vivo studies.

## 9. p53 as an Inducer of Hormones

Interestingly, some of the proteins upregulated by A+N may even exert effects at the systemic level (Figure 4). For instance, METRNL (subfatin) can stimulate some immune cells to infiltrate adipose tissue and activate their prothermogenic action [49]. *ENHO* encodes adropin, a peptide hormone that plays a critical role in glucose and lipid metabolism, as well as insulin resistance. Additionally, it influences immune cell function and inflammation, exerting anti-inflammatory effects [50]. Another particularly interesting protein encoded by a gene upregulated by A+N is GDF-15. It is also strongly upregulated by camptothecin. This gene was already found to be activated by p53 [51]. Recently, in a mouse model of lung cancer, tumour-derived GDF-15 was found to be responsible for cancer-associated cachexia [52]. GDF-15 is an endocrine factor that acts on receptors localised in the brain and transmits metabolic signals leading to weight reduction effects by directly reducing appetite [53]. This weight loss-inducing function of GDF-15 can be abrogated by ponsegromab, which is being considered as a new miracle drug for cancer-related cachexia [54]. Malnutrition is a severe problem among most cancer patients, especially those with pancreatic cancer [55].

It is very likely that in cancers with wild-type p53, GDF-15 upregulated by therapeutic agents may augment food aversion (which is usually already present in cancer patients) and other physiological changes leading to cachexia. Moreover, the systemic administration of many anticancer drugs also activates p53 in normal cells, hence they could possibly be the source of GDF-15. In an initial study, the authors demonstrated that GDF-15 activated by p53 promotes apoptosis [51]. However, there is another possibility that p53, by activating this gene, can influence the functioning of the whole organism [53]. Food aversion induced by p53 is biologically plausible. Exposure to DNA-damaging agents, such as ionising radiation or genotoxic substances, frequently leads to the destruction of the epithelia lining the digestive system. Under these stressful conditions, the activated p53 may lower food intake by inducing GDF-15, which helps in healing the damaged epithelia.

Interestingly, p53 activates the *GAST* gene, which encodes the hormone gastrin [10]. Thus, p53 not only influences stressed cells and their immediate environment, but may also affect distant organs by promoting the secretion of peptide hormones like GDF-15, gastrin, and probably many others. This plausible role of p53 in the functioning of the endocrine system is poorly studied, although it is well known that germline mutations of p53 significantly increase the risk of a cancer developing in a hormone-producing tissue—the cortex of the adrenal glands [56]. Analysis of transcriptomic data [10] shows that p53 may also promote the expression of proteins, e.g., CPE (carboxypeptidase E), that participate in the maturation and secretion of protein hormones including proinsulin and proenkephalin from secretory vesicles [57]. Thus, there is a possibility that p53 not only promotes the production of secretory proteins but also molecules that participate in their maturation and exocytosis.

## 10. p53 Activation by Actinomycin D and Nutlin-3a—Physiology or Artefact?

One major question that permeates research utilising the combination of actinomycin D and nutlin-3a concerns the physiological significance of the results. After all, cells are not physiologically exposed to these substances. On the other hand, many p53 functions have been discovered by exposing cells to anticancer drugs, which are also not natural activators. This raises an important question: does this mode of p53 activation resemble a naturally occurring stress factor? It is plausible that exposure to actinomycin D and nutlin-3a may resemble stress associated with bacterial infections. One has to keep in mind that both actinomycin D and nutlin-3a are xenobiotics. Actinomycin D is also a bacterial metabolite. There may be mechanisms that are capable of sensing various molecules produced by microorganisms and activating p53 as part of the innate immune response. We base this hypothesis on the observation that several genes upregulated by A+N encode proteins with antibacterial functions, e.g., DEFB1, LACC1, and WFDC2 (reviewed by Łasut-Szyszka et al. [58]). On the other hand, if this system is artificial, the important question is what lessons can be learned from using it. The data generated by exposing cells to A+N have provided valuable information that can inspire many interesting hypotheses for further investigation. Some of these hypotheses have been discussed here. Moreover, these findings may have potential clinical applications. This treatment modality strongly sensitises cells to apoptosis induced by at least one death receptor agonist (FASLG). But do the new p53-regulated genes identified in cells exposed to A+N represent the genuine p53 targets that can also be activated under specific, naturally occurring stress conditions? We suspect that many genes strongly activated by A+N in A549 cells can also be activated in other cell types exposed to less extreme stress factors. An interesting example is the *DUSP13* gene. A+N stimulate *DUSP13* expression from an alternative, p53-responsive promoter, resulting in the production of a DUSP13 isoform whose function remains unknown. This DUSP13 variant appears in A549 cells as a result of the p53-dependent apparently synergistic activity of actinomycin D and nutlin-3a, but in other cell lines, this isoform is expressed following exposure to camptothecin or nutlin-3a alone [59]. Another example is the upregulation of the *TREM2* gene, which is associated with a risk of Alzheimer’s disease [60]. Actinomycin D and nutlin-3a strongly cooperate in activating its expression in the A549 and A375 cell lines. However, in U-2 OS cells, this gene is strongly stimulated by nutlin-3a alone [22]. It is possible that in U-2 OS cells, TREM2 is located within open, transcriptionally active chromatin and the “standard” activation of p53 by nutlin-3a is enough to activate its expression, whereas in other cell types, the chromatin around TREM2 is more compact, requiring a stronger activation of p53 to drive its expression. Generally, the results of RNA-Seq of cells exposed to A+N show that many genes strongly activated by this drug combination are also activated either by actinomycin D or by nutlin-3a, but to a lesser degree (Figure 2). Thus, genes activated by A+N in one particular cell line can be considered strong candidate p53 target genes that warrant validation in cells derived from other tissues. In other words, generating superactivated p53 by treatment with A+N may provide a useful strategy for uncovering p53 targets. Moreover, many p53 target genes could have been overlooked in previous high-throughput searches because the commonly employed cell lines (e.g., HCT116, U-2 OS) harbour gain-of-function mutations in the *PPM1D* gene, which is a negative regulator of p53 [61].

One cautionary note is that the genes identified as being activated by A+N should be tested in isogenic p53-proficient and p53-deficient cells before being classified as p53 targets. This validation is indispensable because some genes, e.g., *IFIT2*, can be activated by this combination independently of p53 [20]. However, genes such as *IFIT2* are rare exceptions [10,19,20].

## 11. Other Examples of p53 Activation by Drug Combinations

The use of drug combinations that collaborate to activate p53 is a legitimate approach in p53 research and preclinical experiments. For instance, David Lane’s group tested various combinations of drugs to determine whether they collaborate in the activation of p53 [62]. Their results showed that low doses (1 nM) of actinomycin D induce a p53-dependent effect on cells similar to that elicited by 4 μM nutlin-3. Subsequently, they combined either actinomycin D or nutlin-3a with various cytotoxic drugs used in cancer treatment (melphalan, etoposide and doxorubicine) and tested whether these combinations collaborate in the activation of p53. Apparently synergistic effects were observed between melphalan and nutlin-3a, etoposide and nutlin-3a, and doxorubicine and nutlin-3. Moreover, they noticed synergy between melphalan and actinomycin D and between etoposide and actinomycin D. Intriguingly, doxorubicin did not synergise with actinomycin D in p53 activation. It would be interesting to find out if these combinations also synergise in sensitising cells to the FAS ligand. Curiously, Choong et al. [62] did not test actinomycin D and nutlin-3a in combination; however, this combination was used by Miyachi et al. [63] against rhabdomyosarcoma cells, resulting in a strong induction of apoptosis.

An extensive review of the effects of nutlin-3a was presented by Kocik et al. [64]. One of the major limitations of nutlin-3a as a potential anticancer drug is its low ability to induce apoptosis or other forms of cancer cell death when used alone, hence it must be combined with other agents to be effective in killing cancer cells. The authors presented an extensive list of various combinations of nutlin-3a or alternative MDM2 antagonists with other compounds or ionising radiation. Some of these combinations were only tested in vitro, others progressed to in vivo animal studies, but only a few reached clinical trials, most of which were Phase I studies involving patients with acute myeloid leukaemia. As mentioned above, in our experience, actinomycin D or etoposide combined with nutlin-3a or other MDM2 antagonists produce striking, p53-dependent sensitisation of cancer cells to the proapoptotic activity of the FAS ligand. Unfortunately, so far, the MDM2 antagonists either acting alone or in combinations with other molecules did not live up to expectations. Despite these limitations, e.g., significant thrombocytopenia emerging as a dose-limiting toxicity linked to prolonged Mdm2 inhibition, some MDM2 inhibitors reached Phase II clinical trials against tumours with wild-type p53 [65]. To update this information, we searched the ClinicalTrials.gov website (21 September 2026) and found that idasanutlin (RG7388) is not currently included in any active trials. Fifteen trials involving this compound were either completed, terminated, or withdrawn. AMG 232 (navtemadlin) had been investigated in six trials that had been either completed or terminated. Currently, it is being investigated in two active trials, both Phase I studies, against acute myeloid leukaemia. HDM201 had been investigated in 12 trials (terminated, completed, withdrawn). Currently, it is being investigated in one active trial (Phase I/II) in combination with Pazopanib (tyrosine kinase inhibitor), against advanced/metastatic soft tissue sarcoma with wild-type p53. Additionally, one study involving HDM201 was in the process of recruiting patients. This Phase II trial involves various targeted therapy drugs and different types of solid tumours. Another MDM2 antagonist, APG-115, is currently being investigated in five studies that were recruiting patients (Phase I, Phase I/II, or Phase II). Thus, some MDM2 inhibitors are currently being investigated in clinical trials (Phase I or Phase II), mostly against haematological malignancies but also against solid tumours (e.g., sarcomas, melanomas, or other tumours). Hopefully, an MDM2 antagonist, likely in combination with another drug, will ultimately reach clinical application.

Another approach employing p53 involves reactivating the nonfunctional protein using compounds directed against specific mutations [66]. A recent clinical trial involving such a compound gave promising results [67].

There are other examples of drug combinations that collaborate in the activation of p53 and induction of p53-dependent biological outcomes. For instance, inauhzin inhibits SIRT1, which is a deacetylase of p53. The inhibitor promotes the acetylation of p53, which is an important step in its activation [7]. The combination of inauhzin with nutlin-3a synergise in p53 activation and in the growth inhibition of cancer cells, the induction of apoptosis, and in the reduction in tumour growth in vivo [68].

One of the first stress factors found to promote p53 phosphorylation at its amino-terminus was ionising radiation (IR). Hence, if the model presented in Figure 1 is correct, one would expect that IR and MDM2 antagonists would also synergise in the activation of p53 and the promotion of p53-dependent biological outcomes. Vargas et al. [69] used endometrial cancer cells as a model. The cells with wild-type p53 were exposed to one of two MDM2 antagonists, nutlin-3a or AMG-232. The cells were also treated with IR. In vitro experiments demonstrated that nutlin-3a synergised with IR in one of the examined cell lines, whereas AMG-232 synergised with IR in both cell lines. The synergy was also evident in an in vivo xenograft model involving treatment with AMG-232 and IR. In this study, the word “synergy” was used in a strict mathematical sense because the authors calculated its degree using dedicated mathematical models. Moreover, a novel MDM2 antagonist, APG-115, enhanced the radiosensitivity of colorectal cancer cells both in vitro and in vivo. At the molecular level, the combination of APG-115 and radiation markedly enhanced activation of the p53 pathway in cell lines and xenograft tumour tissues [70].

Interesting data were published by Fang et al. [71]. These authors demonstrated that the MDM2 antagonist APG-115 collaborated with PD-1 blockade in a syngeneic mouse tumour model. This effect was visible regardless of the p53 status of the cancer cells (wild-type, mutant, or null) but was abolished in host mice with p53 knockout. Thus, p53 activity of non-cancerous cells in the tumour microenvironment may significantly influence the ability of the immune system to eliminate cancer cells.

The synergistic (in the strict sense) effect between APG-115 and various standard-of-care agents was detected in preclinical models (both in vitro and in vivo) of acute myeloid leukaemia. However, the authors present a different hypothesis explaining the synergy. They propose that APG-115 and standard-of-care agents augment leukaemia cell killing by complementarily activating the p53/p21 pathway and upregulating DNA damage [72].

Other drug combinations do not synergise in p53 activation itself but rather in the induction of p53-dependent biological effects. For instance, nutlin-3a collaborates with navitoclax (an inhibitor of antiapoptotic proteins BCL-XL/BCL2) in the killing of acute lymphoblastic leukaemia cells both ex vivo and in xenograft models [73]. This type of leukaemia frequently retains wild-type p53 and may therefore represent a suitable disease context for drug combinations aimed at activating p53.

Above, we presented examples of drug combinations other than A+N that collaborate in the activation of p53. Here, we present several examples of drug combinations whose effects vary depending on p53 status. Considering the important role of p53 in DNA protection [7], we chose to examine the role of p53 status in the modulation of cancer cell sensitivity to PARP1 (Poly ADP-Ribose Polymerase 1) inhibitors (PARPis). This enzyme participates in the detection of single-strand breaks in DNA and modifies nearby chromatin, thereby attracting other DNA repair enzymes that directly seal the breaks. Moreover, PARP1 participates in other forms of DNA maintenance, such as nucleotide excision repair, double-strand break repair, and the stabilisation of DNA replication forks. Inhibition of this enzyme preferentially kills cancer cells with impaired DNA break repair due to mutations in *BRCA1* or *BRCA2* genes. Thus, PARPis are used to treat cancers (frequently breast cancers) with mutations in these genes [74]. Because p53 promotes DNA repair [7], researchers expected p53 to have a protective role against PARPi-mediated cytotoxicity. However, the relationship between p53 status in cancer cells and their sensitivity to PARPis is not straightforward. For instance, enhanced cytotoxicity was observed when PARPi was combined with Pifithrin α, a drug that inhibits p53. Unexpectedly, increased sensitivity to PARPi swas also observed in cells exposed to nutlin-3a, which activates p53. As a mechanistic explanation, the authors suggest that this may be caused by nutlin-induced repression of RAD51, which participates in DNA repair by homologous recombination [75]. Other authors, using a different experimental model (breast and glioma cells with proficient homologous recombination repair), found that p53 is required for PARPi sensitisation by ionising radiation [76]. In a more recent study performed using patient-derived tumour organoids (breast or lung cancers), the combination of temozolomide (an alkylating agent) and PARPi was evaluated. The combination of the two drugs was synergistic for a cytotoxic response in the organoids with mutant p53 but not in organoids with wild-type p53. The combination of PARPis and temozolomide induced more DNA double-strand breaks in mutant p53 expressing organoids than in wild-type p53 expressing organoids. These results, obtained using material closely resembling clinical settings, suggest that p53 mutations could serve as a biomarker for predicting the response to combined PARPi (talazoparib)-temozolomide treatment, and are consistent with the hypothesis that p53 protects against PARPi-mediated cytotoxicity [77]. A similar conclusion concerning p53 was reached in experiments using colorectal and pancreatic cancer cell lines treated with combinations of trifluorothymidine and PARPis, which showed superior anti-neoplastic activity in mutant p53 cancer models [78]. Another very interesting drug combination involves the purported reactivator of mutant p53 (HO-3867) and PARPi olaparib. HO-3867 acted synergistically with olaparib treatment in five cell lines studied in vitro as well as in vivo in a xenograft model of high-grade serous ovarian cancer. However, it should be kept in mind that HO-3867 acts through both p53-independent and p53-dependent pathways, resulting in inhibition of homologous recombination-mediated DNA repair in p53-mutant cancer cells [79]. Thus, the collaboration between olaparib and HO-3867 could result from inhibition of DNA repair by the latter compound.

## 12. Concluding Remarks and Outlook

Our own experience indicates that the best way to superactivate p53 is to use one factor that triggers the phosphorylation of the p53 transactivation domain and another factor that antagonises the MDM2 protein. The best-studied pair of compounds is actinomycin D (inducer of phosphorylation) and nutlin-3a (the antagonist of MDM2). The molecular mechanism underlying this cooperation is not precisely known. The hypothesis is that preventing the interaction between p53 and MDM2 gives various kinases an opportunity to extensively phosphorylate the TAD of p53, thereby promoting its interaction with transcriptional co-activators like p300 [13]. This hypothesis can be used to explain biological effects of other pairs of compounds like etoposide and nutlin-3a or actinomycin D and RG7112, which cooperate to sensitise cancer cells to FASLG-induced apoptosis [31]. Interestingly, collaboration between etoposide and nutlin-3 in the activation of p53 was also observed by others [62], who additionally noticed a combined effect of doxorubicine and nutlin-3a. The mechanism of cooperation between these compounds can also be explained by the aforementioned hypothesis, because doxorubicine is known to induce ATM-dependent phosphorylation of p53 on Ser6, Ser9, Ser20, Ser37, Ser46 and Ser392 [80]. However, this hypothesis cannot elucidate the collaboration between melphalan and actinomycin D or between etoposide and actinomycin D [62]. Hence, mechanisms other than those presented above are also possible. Regardless of the mechanism, superactivated p53 can be used to identify previously unknown p53 target genes or to test new therapeutic strategies.

If the history of cancer research has taught us anything, it is that no single agent will provide sufficient efficacy as a powerful anticancer drug. Even in cancer cells with wild-type p53, its full proapoptotic potential can be unleashed only after the combined action of three agents, e.g., actinomycin D, an MDM2 antagonist, and a death receptor agonist [31]. Thus, identifying drug combinations, particularly those with synergistic effects, remains a high priority. There are new bioinformatic approaches that may accelerate the identification of synergistic drug combinations by integrating high-throughput drug screening with gene-expression profiling and other molecular data [81]. Such innovative methods are needed to address the enormous number of drug combinations that are possible, arising from the large number of various substances that are currently available for treatment. Some combinations can be deduced based on the known molecular mechanisms of drug actions, as exemplified by the combination of actinomycin D or similar agents with MDM2 antagonists. However, other combinations may be less obvious but potentially effective. Current tools combining various “omics” together with novel tools in computer science, including artificial intelligence, offer a promising framework for developing more effective treatments for cancer and other diseases [82].

## Figures and Tables

**Figure 1:**
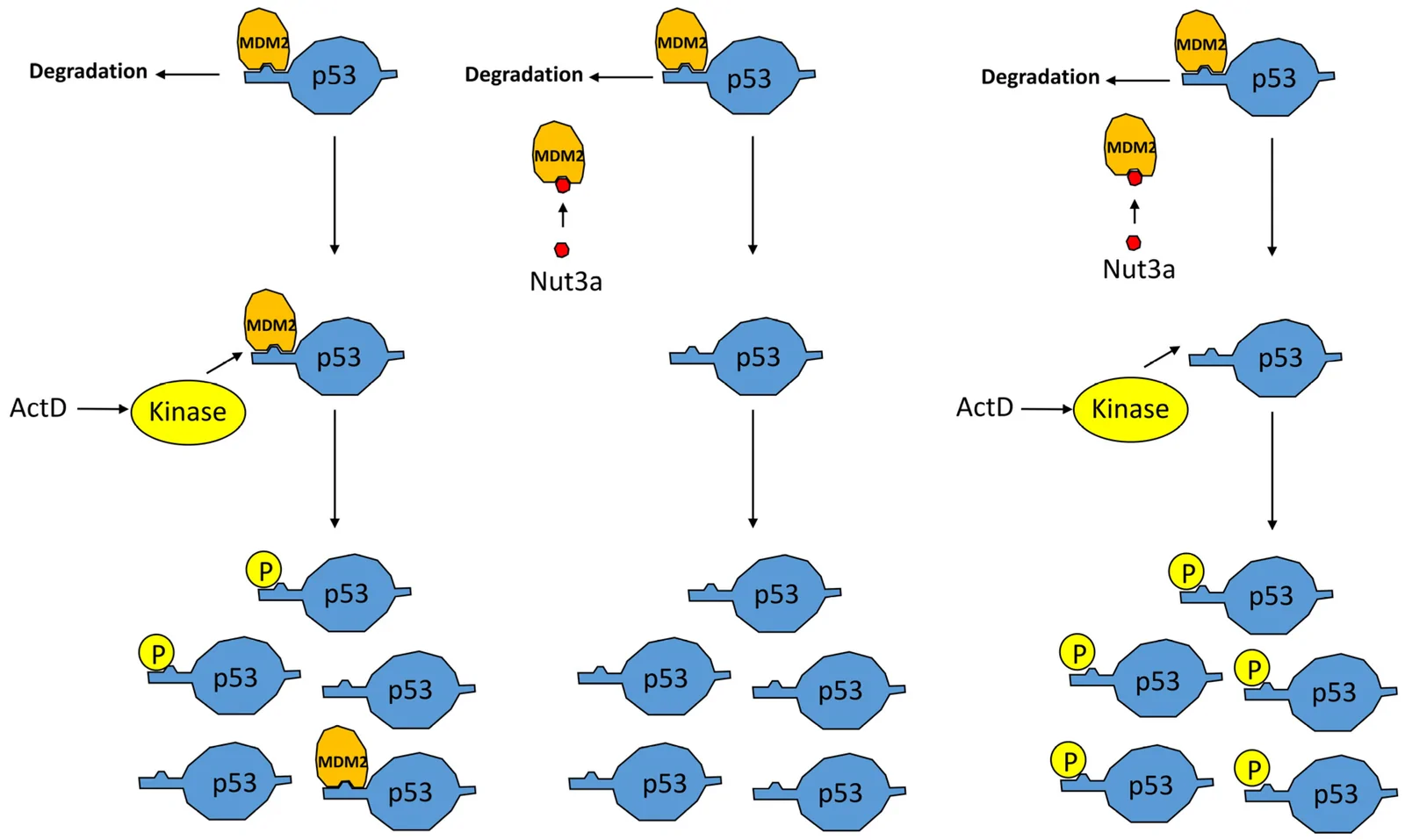
The hypothetical mechanism whereby actinomycin D (ActD) and nutlin-3a (Nut3a) collaborate in the activation of p53. The MDM2 protein is the major negative regulator of p53. MDM2 specifically binds to the N-terminal fragment of p53, concealing its transcription-activating domain and promoting p53 degradation. In cells exposed to stress factors, including actinomycin D (ActD), various kinases are activated that phosphorylate amino acids on the N-terminal fragment of p53, thereby preventing interaction between MDM2 and p53, which blocks its degradation and creates docking sites for various transcription regulators cooperating with p53. However, to phosphorylate p53, the kinases must compete with MDM2. This is likely why only a limited number of p53 molecules are phosphorylated; however, this is enough to stabilise some p53 molecules and activate p53 target genes. Moreover, stress kinases also phosphorylate and inactivate MDM2, which contributes to p53 activation [17] (not shown here). Some p53 molecules remain unphosphorylated, bound to MDM2, and rendered unstable. In cells exposed only to nutlin-3a, p53 is stabilised because MDM2 is prevented from targeting p53 for degradation, but p53 is not extensively phosphorylated as is shown in experiments with A549 cell line [18]. Interestingly, in other cell lines, e.g., NCI-H28, increased expression of phosphorylated p53 molecules can be detected also in cells exposed to nutlin-3a alone; however it is not as large as in cells exposed to A+N [18]. In cells exposed to both actinomycin D and nutlin-3a, MDM2 does not conceal the N-terminal fragment of p53 and the kinases have easy access to the protein, resulting in extensive phosphorylation of its molecules. Why actinomycin D and nutlin-3a also promote the phosphorylation of p53 on its carboxyl terminus, e.g., Ser392, is not clear.

**Figure 2:**
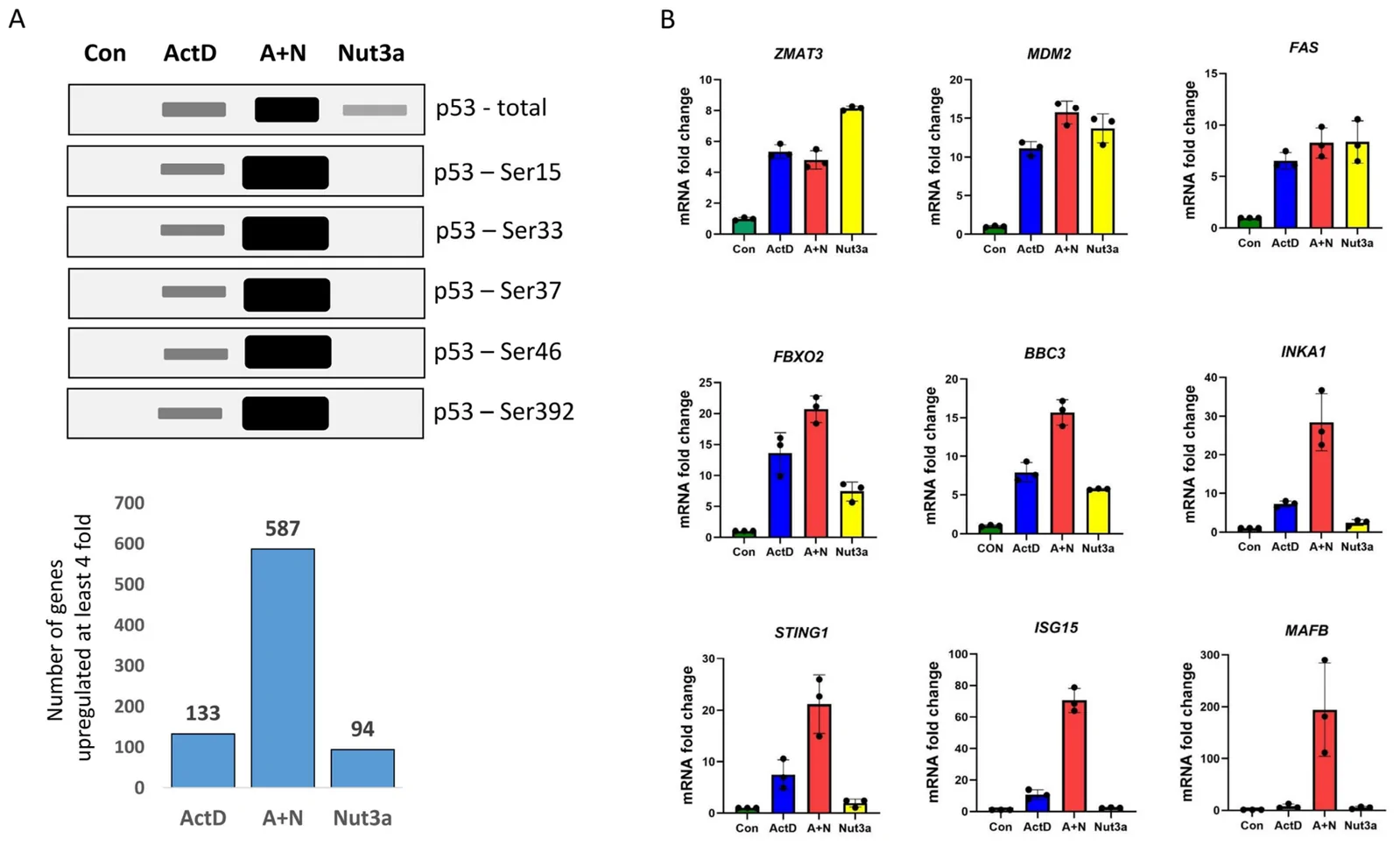
Schematic representation of synergy between actinomycin D (ActD) and nutlin-3a (Nut3a) in the phosphorylation of p53 and upregulation of p53 target genes. (**A**) The depiction of Western blotting results showing the expression of total p53 and p53 with phosphorylated serines 15, 33, 37, 46 and 392 in A549 cells exposed to actinomycin D, nutlin-3a, or to both substances (A+N) (based on the data presented in [10,18,19,20]). The lower panel shows the number of genes upregulated at least 4-fold in A549 cells exposed as shown (based on [10]). (**B**) The examples of established p53 target genes and a putative p53 target (*INKA1*) showing various responses to the treatment with actinomycin D and nutlin-3a. The graphs were prepared using GraphPad Prism (10.6.1) based on the RNA-Seq data published by Łasut-Szyszka et al. [10].

**Figure 3:**
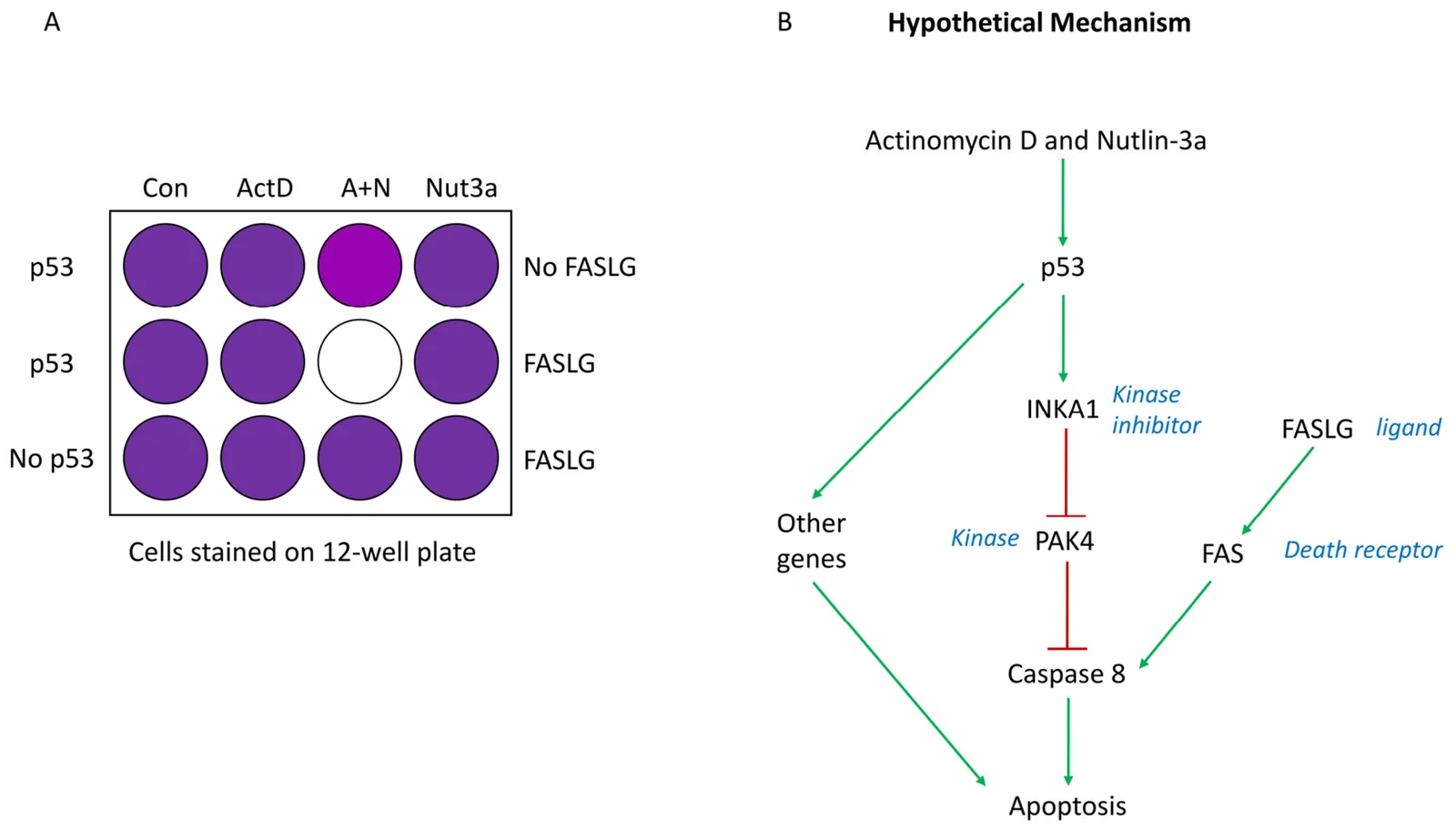
The p53 activated by actinomycin D and nutlin-3a strongly sensitise cancer cells to FAS ligand (FASLG)-induced death. (**A**) Estimation of cell death and survival using cell staining on culture plates. The purple circle represents the cell monolayer. Treatment with A+N inhibits cell growth, which is visible as slightly lighter staining. However, when FASLG is added, most cells die and cell population does not regrow; hence, there is an absence of staining (white circle). In this assay, actinomycin D or nutlin-3a alone have no major effect; only the synergy of these substances induces sensitisation to FASLG. When an isogenic p53-deficient cell line is treated, the sensitising effect is completely lost, showing complete dependence on wild-type p53 (based on [31]). (**B**) Hypothetical mechanism of cell sensitisation to FASLG induced by p53 activated by A+N. p53 likely induces expression of the *INKA1* gene, which encodes a kinase inhibitor that negatively regulates PAK4 kinase [32]. This enzyme in turn inhibits caspase 8, a major caspase activated by death receptors [33]. This signalling pathway has been demonstrated experimentally; however, its contribution to the sensitising effect in this particular experimental setting remains to be demonstrated, as p53 upregulates dozens of other apoptosis-related genes, which may contribute to the sensitising effect, sometimes in surprising ways.

**Figure 4:**
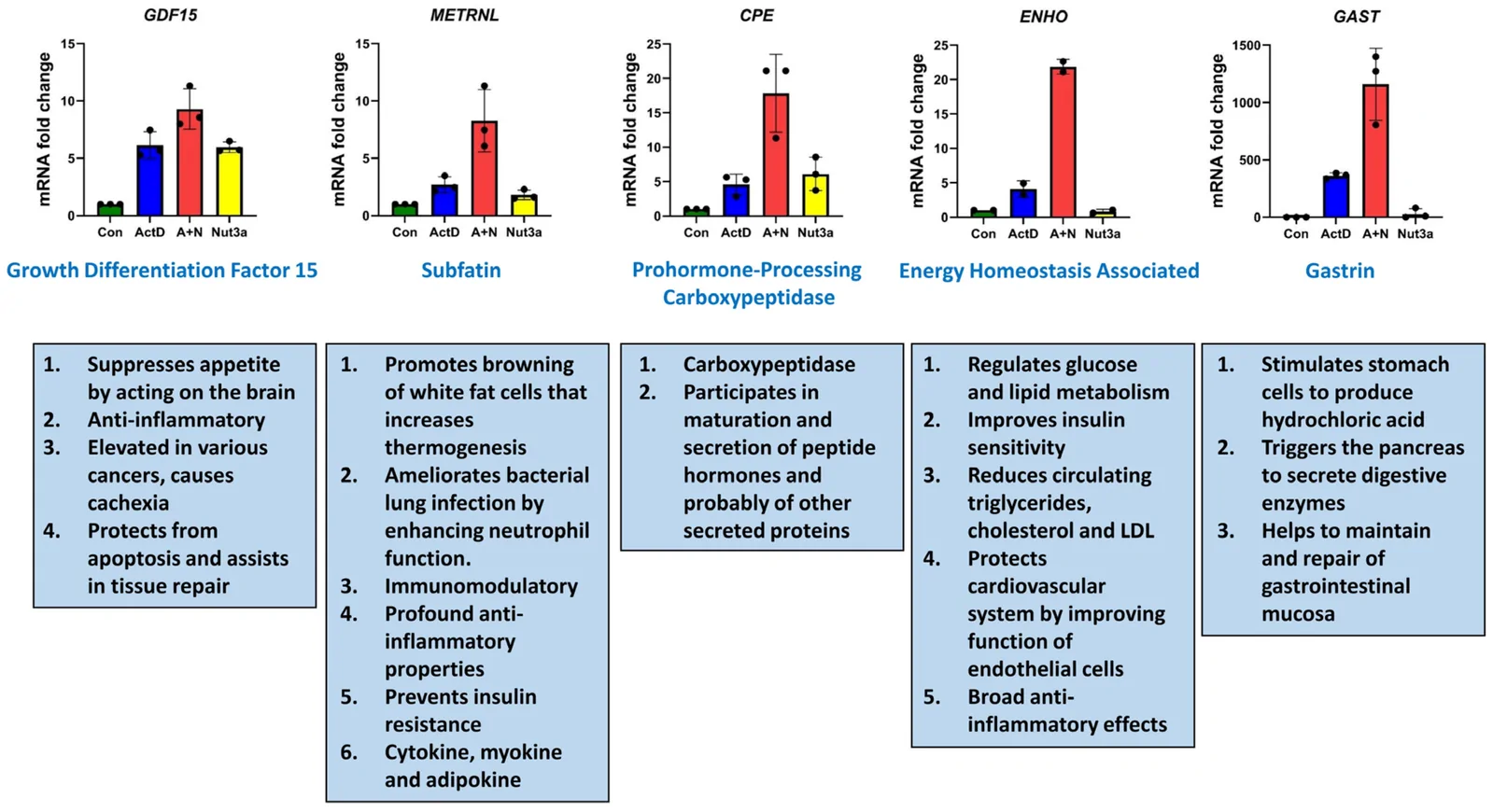
A549 cells exposed to actinomycin D and nutlin-3a, either alone or in combination (A+N), secrete hormones or hormone-related molecules (CPE). The graphs show the fold changes in mRNA levels of the indicated genes based on data published in [10]. The lower panels summarise the functions of the proteins. *GDF15* and *GAST* were found to be regulated by p53 [10,51].

## Data Availability

No new data were created or analyzed in this study. Data sharing is not applicable to this article.
